# A chemometric approach to assess the oil composition and content of microwave-treated mustard (*Brassica juncea*) seeds using Vis–NIR–SWIR hyperspectral imaging

**DOI:** 10.1038/s41598-024-63073-0

**Published:** 2024-07-08

**Authors:** Rajendra Hamad, Subir Kumar Chakraborty

**Affiliations:** Agro Produce Processing Division, ICAR-Central Institute of Agricultural Engineering, Beraisa Road, Nabibagh, Bhopal, 462038 India

**Keywords:** Energy science and technology, Engineering

## Abstract

The wide gap between the demand and supply of edible mustard oil can be overcome to a certain extent by enhancing the oil-recovery during mechanical oil expression. It has been reported that microwave (MW) pre-treatment of mustard seeds can have a positive effect on the availability of mechanically expressible oil. Hyperspectral imaging (HSI) was used to understand the change in spatial spread of oil in the microwave (MW) treated seeds with bed thickness and time of exposure as variables, using visible near-infrared (Vis–NIR, 400–1000 nm) and short-wave infrared (SWIR, 1000–1700 nm) systems. The spectral data was analysed using chemometric techniques such as partial least square discriminant analysis (PLS-DA) and regression (PLSR) to develop prediction models. The PLS-DA model demonstrated a strong capability to classify the mustard seeds subjected to different MW pre-treatments from control samples with a high accuracy level of 96.6 and 99.5% for Vis–NIR and SWIR-HSI, respectively. PLSR model developed with SWIR-HSI spectral data predicted (R^2^ > 0.90) the oil content and fatty acid components such as oleic acid, erucic acid, saturated fatty acids, and PUFAs closest to the results obtained by analytical techniques. However, these predictions (R^2^ > 0.70) were less accurate while using the Vis–NIR spectral data.

## Introduction

Mustard (*Brassica juncea*) is a nutritionally rich crop that contains a lot of minerals and a high-quality of edible oil. The seeds of mustard which contain an average of 38.3% oil, are grown in many countries for the production of vegetable oil^1^. Mustard oil is the most important and widely used edible oil for several cuisines of the world. The fatty acids present in mustard oil are known to have positive effects on heart health and skin health, as well as anti-inflammatory properties^2,3^. All the said reasons have been the cause behind a higher demand for this oil.

Despite the growing demand, there remains a gap between production and supply^4^. Besides the limited production of mustard seeds, contributing to the low availability of mustard oil is the low recovery rate of oil from the seeds in the oil mills, which is only 26–27%. The improvement of recovery rates is vital to meet the growing demand and ensure that consumers can benefit from the numerous health benefits of mustard oil^5^. One way of enhancing the millable oil from mustard seeds can be by implementing different oil extraction processes and subjecting the seeds to pre-treatments that will increase the yield of oil. The implementation of these processes will not only benefit the consumers by ensuring a steady supply of high-quality mustard oil, but also the producers, as they can maximize the use of raw materials and better the economics of the process.

The most common methods for the extraction of vegetable oil are milling (mechanical pressing) and solvent extraction^6^. While solvent oil extraction is currently the most effective method, there are several technological drawbacks to this technique. These include high operational costs and low-quality products due to the high temperatures required during processing, food safety issues, and emissions of volatile organic compounds into the environment^7^. Oil extracted by mechanical pressing is significantly less labour-intensive and less expensive than the solvent extraction technique^8^. Compared to the more sophisticated solvent extraction machinery, the mechanical pressing method has the distinct advantage of being both safe and easy to implement. Besides this milling of materials have several benefits including the preservation of the material’s inherent qualities, the absence of chemicals in the final product and a safe procedure. Nowadays, people are becoming more concerned about their health as their living conditions rise; as a result, healthy and nutritious food products are becoming more popular; sometimes even at a premium price. As a result, there is an increasing market demand for milled oil in most developed and developing countries.

However, a major drawback of mechanical milling is that a substantial amount of the oil cannot be recovered and it remains in the milled cake. To overcome this problem a number of operations have been coupled with mechanical oil extraction; size reduction, grinding, dehulling, breaking, heat treatment (cooking), and enzymatic hydrolysis are examples of traditional pre-treatment techniques used to weaken the cellular material for an enhanced oil extraction ratio^9^. However, to better overall yield, researchers are looking into certain cutting-edge alternative pre-treatment technologies. Among these pre-treatments, infrared, microwaves (MW) and ohmic heating have received a considerable amount of research attention. These innovative methods can enhance the oil recovery as well as the nutritional value, physicochemical characteristics, and sensory qualities of the extracted oil^10^.

Over the period of time, MW pre-treatment has gained popularity as an enhanced oil recovery pre-treatment tool for various industrial applications due to its numerous advantages over traditional heating methods. The MW technology uses microwave radiation energy to directly heat the material, resulting in uniform and quick heating throughout the whole volume of the material^11,12^. This is achieved by molecular interaction between the electromagnetic field and the material, leading to heat generation. MW pre-treatment offers specific internal heat generation proportional to the moisture content of the material. This ensures rapid and uniform heating without the presence of thermal gradients, making the process more efficient and precise^13^. The uniform heating of the material results in shorter processing times and energy savings, making it a more efficient and cost-effective method of pre-treatment compared to other heating methods. MW pre-treatment on mustard seeds conducted prior to oil extraction, causes a rupture of the cell membrane, leading to an increase in oil yield. The permanent pores created by MW treatment deep down at the central core of the seed allow the oil to flow freely through the cell membrane, resulting in a higher oil extraction rate^14^.

Spectroscopy and Hyperspectral imaging (HSI) techniques have been widely adopted for the chemical-free, rapid and non-destructive evaluation and measurement of the chemical composition of the various products in the food industry. HSI, in particular, offers several advantages over other techniques such as NIR spectroscopy, multispectral imaging, and conventional RGB imaging. These advantages include the capability to gather spatial and spectral data as well as heightened sensitivity for even the smallest components^15^.

HSI has been successfully used for the determination of oil content and fatty acid content in various oil seed crops^16^. It has been reported that this method has been used to accurately estimate the oil content and fatty acid traits in soybean^17^, peanut kernel^18^, rapeseed^19^, and also effective for the simultaneous measurement of major components^20^. However, few researchers have also reported the quantification of oil content and fatty acid contents in *Brassica* seeds by using HSI^21,22^.

HSI generates a huge volume of data, and multivariate analysis methods are frequently applied to find the relevant information from this data^23^. Principal component analysis (PCA) is frequently utilized to obtain a preliminary understanding of the HSI spectral data, while other chemometric methods such as partial least squares discriminant analysis (PLS-DA) and regression (PLSR) are used for classifying, predicting, and quantifying specific parameters in sample data^24,25^. Furthermore, the application of variable selection methods can help identify the most meaningful spectral areas and eliminate any redundant information from the spectrum. By utilizing these techniques, HSI proves to be an effective tool in the analysis and evaluation of food products, providing valuable insights into their chemical composition and quality.

Therefore, the aim of this study was to estimate the change in mechanically expressible oil in terms of its availability and fatty acid profile for microwave-treated mustard seeds. While, Vis–NIR–SWIR hyperspectral imaging was used to visualize the spatial information for the spread of oil in the ruptured mustard seeds after the microwave treatment; the HSI spectral data would be used for development of prediction models for oil and fatty acid content.

## Materials and methods

### Mustard seed

Mustard seeds (*Brassica juncea*) of variety RH-0749 were purchased from the National Seed Corporation, Bhopal, India. The moisture content of the seed was 7 (± 0.02)% and its average diameter was 1.6 ± 0.4 mm. Seeds were cleaned of all the foreign material before being stored safely in sealed pouches at 4 °C. They were accessed as per the experimental requirement and allowed to equilibrate before being used further.

### Microwave pre-treatment

The mustard seed samples were treated in a microwave oven (CE1111TL, Samsung, India) operating at a frequency of 2.45 GHz. The microwave oven had a power range of 0 to 800 W. Mustard seeds were taken in batches of 250 g for each microwave (MW) pre-treatment experiment and placed in a glass container. Three different MW power levels (180, 300 and 450 W), time of exposure (120, 240 and 360 s) and bed thickness (5, 10, and 15 mm) were selected as per full factorial experimental design for each pre-treatment. Similar pre-treatment has been reported for oil production from palm^26^ and Chilean hazelnuts^27^. The MW-treated samples from the three replicates were collected and allowed to cool at room temperature before the oil was extracted from the mustard seeds by single screw mechanical oil expression. Based on preliminary experiments, two (MW1 and MW2) microwave treatment conditions in terms of time of exposure and bed thickness of mustard seeds inside microwave oven at a fixed power level of 450 W were selected for the classification and quantification of oil and fatty acid content using HSI. The experimental conditions for MW1 and MW2 in terms of time of exposure and bed thickness were 240 s, 5 mm and 360 s and 15 mm respectively.

### Oil extraction

The oil from the mustard samples was obtained by milling both MW treated and untreated mustard seeds with a laboratory-scale oil expeller (SH-400, Shreeja Health Care Products, India). The milled crude oil was first filtered through a sieve to remove large impurities and then through a muslin cloth to remove any tiny suspended particles from the milled oil that may have been produced as a result of the milling operation. After that, the oil that had been extracted was collected for analysis in polyethene terephthalate (PET) bottles, followed by storing at 4 °C in a refrigerator.

### Fatty acid profiling

The fatty acids present in mustard oil samples were identified after the triglycerides were converted to methyl esters using methanol and boron trifluoride as a catalyst^28^. The resulting fatty acid methyl esters (FAMEs) were analysed using gas chromatography-mass spectrometry (GC–MS/MS, GC-2010, Shimadzu Corporation, Japan). The system included an AOC-20i + s chromatograph interfaced with a QP 2010 Ultra mass spectrometer and was equipped with an ELITE-2560 column (100 m length, 0.25 mm inner diameter, and 0.2 µm film thickness). Helium gas (99.999% purity) was used as the carrier gas at a flow rate of 1.25 mL/min. The initial oven temperature was 100 °C for 4 min, followed by an increase to 240 °C for 15 min with an injection temperature of 225 °C. The mass spectra of FAMEs were compared to NIST library to identify the compounds present, with those showing more than a 90% similarity index being recorded and reported as a relative percentage of the total peak area. The average value of each fatty acid component in both MW-treated and untreated mustard oil samples was obtained, which was used as the reference value for predicting fatty acid composition.

### Hyperspectral imaging (HSI) system

Hyperspectral images of microwave-treated mustard seeds in reflectance mode were obtained, and two HSI systems (Vis–NIR HSI system with a wavelength range of 400–1000 nm and SWIR HSI system with a wavelength range of 1000–1700 nm) were assembled. Vis–NIR HSI system (Specim Spectral Imaging Ltd., Oulu, Finland) has a spectral resolution of 2.8 nm thus acquiring 97 spectral bands. The system was also equipped with three (50W) tungsten halogen bulbs to provide uniform lighting. During imaging, the spectral and spatial binning were set at 8 and 1 (not binned), respectively. SWIR HSI system (Pika NIR-320, Resonon Inc., USA) has the following key characteristics: a spectral resolution of 4.9 mm, spectral bands of 168, spatial channels of 320, and a maximum frame rate of 540 fps. The system also had high-intensity stabilised broadband fibre coupled with a line light source to illuminate evenly while minimising any potential heating effect. To obtain the 3-D hyperspectral data cuboid, the object moved spatially in a second direction over a platform with adjustable speed. For capturing Vis–NIR hyperspectral images, Spectral/DAQ ver. 3.62 software (Spectral Imaging Ltd., Oulu, Finland) and for capturing SWIR hyperspectral images, Spectronon Pro ver. 3.4.5 (Resonon Inc., Bozeman, MT, USA) image-capturing software was used to control the imaging conditions, i.e., binning, integration time, and sample movement. All procedures and methods conducted throughout this study adhered to the institutional guidelines and regulations.

### Data extraction

During the analysis, the reflectance values of all pixels were individually obtained for each MW treated mustard seeds. The spectral data of all pixels within each seed sample belonging to different treatments were obtained. Each test sample for HSI comprised 78 (± 8) mustard seeds from which 900 spectral reflectance values were collected and utilized for the development of models. In total, 2700 pixel data of Vis–NIR with 97 wavelengths and 2700 pixel data of SWIR hyperspectral images with 137 wavelengths were collected for analysis. To ensure high prediction accuracy and minimize errors, the dataset was divided into separate training (calibration and cross validation) and testing sets using random sampling. The training set consisted of 70% (630 spectra) of each sample, while the testing set comprised 30% (270 spectra) of each sample.

### Pre-processing of hyperspectral images

The spatial pre-processing of hyperspectral images was a prerequisite for subsequent image processing. The purpose of spatial pre-processing was to reduce noise, eliminate distortions caused by geometric variations, improve the data accuracy and eliminate regions of the image that are unusable for multivariate analysis. In this research, the spatial binning approach was used to reduce the spatial dimensions (pixel resolution) of an image followed by the removal of dead pixels. The image background was removed using masking created by the k-means clustering algorithm.

The effects of light scattering and other undesirable factors such as noisy data and variations in the background on the seed morphology were corrected through various pre-processing techniques. Different spectral pre-processing techniques such as Standard Normal Variate (SNV), Savitzky–Golay (SG) Smoothing, SG 1st Derivative (SG-D1) and SG 2nd Derivative (SG-D2) were tested individually and collectively. Both smoothing and derivative were used with 7 point window and a second-order polynomial. Spatial and spectral pre-processing of hyperspectral images was performed using the HYPER-Tools, version 3.0^29^ for MATLAB R2019a^30^ (MathWorks, Natick, USA) environment.

### Multivariate analyses

#### Principal component analysis (PCA)

Principal components analysis (PCA) is a computational data analysis technique that can be used to reduce the dimensionality of the data by identifying and selecting a smaller number of orthogonal components known as principal component (PC), which minimises spectral redundancy while maintaining spatial information^31^. In this study, PCA was used as an exploratory technique on raw and pre-processed data of hyperspectral images to depict the effect of pre-treatments on mustard seeds based on their spectrum variances. PCA analysis was done using the nonlinear iterative partial least squares algorithm (NIPALS)^29^ with mean centring and three PCs. Additionally, hyperspectral images were represented as false colour images by projecting the PCA model to represent pixel colours corresponding to chemical variations^32^.

#### Classification model analysis

The classification models from hyperspectral data were developed with partial least squares discriminant analysis (PLS-DA) to classify the pre-treatments on mustard seeds. These data were split into two sets, with one set containing 70% of the data needed for model development. In order to prevent the model from being overfit, the developed model underwent internal cross-validation using Venetian blind cross-validation with five groups. To validate the created model, the remaining 30% of the data were used as a testing data set. The minimum value of root mean square error of cross validation (RMSECV) with venetian blinds technique was used for selection of the optimum number of latent variables (LV)^33^. In our study, model performance has been statistically validated by the sensitivity (ratio of true positives to actual positives), specificity (data ratio of true negatives to the sum of all negatives), precision (ratio of true positives to all positives), accuracy (ratio of truly identified data to all data), class error and non-assigned rate for the calibration, cross-validation and testing. The given formula was used for the calculations:

1 $$\text{Sensitivity}= \frac{\text{TP}}{(\text{TP }+\text{ FN})},$$

2 $$\text{Specificity}= \frac{\text{TN}}{(\text{TN }+\text{FP}) },$$

3 $$\text{Precision }= \frac{\text{TP}}{(\text{TP}+\text{FP})},$$

4 $$\text{Accuracy}= \frac{\text{TP }+\text{ TN}}{(\text{TP }+\text{ FN }+\text{TN }+\text{FP }) },$$

5 $$\text{Error}= \frac{\text{FP }+\text{ FN}}{(\text{TP }+\text{ FN }+\text{ TN }+\text{FP}) },$$

where TP stands for true positive, TN stands for true negative, FP stands for false positive, and FN stands for false negative.

#### PLSR to predict oil and fatty acid content

To evaluate the effectiveness of hyperspectral data in predicting oil and fatty acid content of microwave-treated mustard seeds, partial least squares regression (PLSR) was used on a calibration set. This method involves developing a linear relationship between X (hyperspectral data) and Y (experimental values) using orthogonal linear combinations of the original variables called latent variables (LV), to maximize the covariance between X and Y^34^. The sample were divided into training (70%) and testing (30%) set. The optimal number of LV was selected using the minimum value of root mean square error (RMSE) and by observing the performance improvement when a new LV was included. The calibration models were built using complete spectra, and the spectra were divided into intervals of variables using the interval PLS (iPLS) method to create iPLS models for each interval and select the best interval of wavelengths^35^. The models were evaluated using the root-mean-square error in calibration (RMSEC), the coefficient of determination (R^2^), and the cross-validation (R^2^_CV_, RMSECV). The models were independently tested using coefficient of determination (R^2^_P_), root of the mean square error for testing (RMSEP), bias (%) and slope.

## Results and discussion

### Oil and fatty acid content

The average oil content measured using AOAC^28^ for untreated mustard seeds was 26.41 (± 0.18) % and MW treated seeds of MW1 and MW2 treatments were 33.71 (± 0.98) and 32.28 (± 0.12) %, respectively; comparable values have been reported for rapeseed oil in the past^12^. The fatty acid composition of (Table 1) MW treated samples as compared with the untreated samples demonstrated an increase of fatty acids like erucic acid (C22:1), linoleic acid (C18:2) and overall monounsaturated fatty acids (MUFA); while a reduction was observed in other fatty acids like alpha-linolenic acid (C18:3), eicosenoic acid (C20:1), oleic acid and polyunsaturated fatty acids (PUFA). Similar observations have been made by other researchers as well^17,36^.

**Table 1:** Oil content (%) and fatty acid content (%) for microwave-treated mustard seeds.

	Untreated	MW treated	
		MW1	MW2
Oil content	26.41 ± 0.18^a^	33.71 ± 0.98^b^	32.28 ± 0.12^b^
Fatty acids			
C18:1	8.88 ± 0.31^a^	7.79 ± 0.25^b^	8.06 ± 0.04^b^
C18:2	14.06 ± 0.34^a^	14.42 ± 0.47^ab^	15.17 ± 0.06^bc^
C20:1	4.89 ± 0.02^a^	4.51 ± 0.13^ab^	4.72 ± 0.03^bc^
C18:3	13.32 ± 0.42^a^	11.43 ± 0.04^b^	12.39 ± 0.42^ac^
C22:1	50.74 ± 0.11^a^	53.63 ± 0.04^b^	51.09 ± 0.11^c^
Saturated	3.18 ± 0.05^a^	3.89 ± 0.04^b^	4.09 ± 0.04^c^
Unsaturated	96.82 ± 0.05^a^	96.11 ± 0.04^a^	95.91 ± 0.04^a^
MUFA	67.25 ± 0.05^a^	68.08 ± 0.90^ab^	66.05 ± 0.10^ac^
PUFA	29.57 ± 0.03^a^	28.03 ± 0.16^a^	29.86 ± 0.17^a^

Treatments do not have a significant difference (p > 0.05) when represented by the same letters (Tukey’s test).

The proportional composition of fatty acids like unsaturated and PUFA in MW treated and untreated remained unaffected significantly (p < 0.05, Tukey’s test), thus it can be inferred that there was no adverse effect on the fatty acid content of the oil due to microwave treatment of mustard seeds.

### Spectral profile

**Figure 1:**
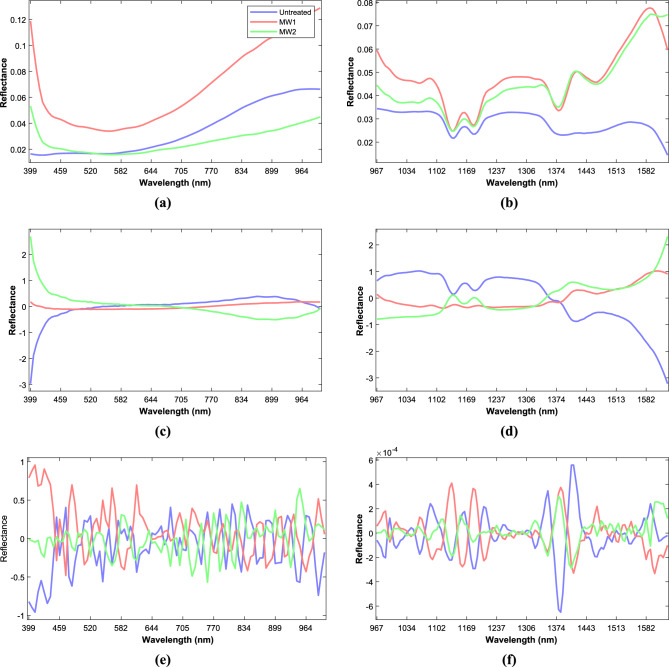
Mean spectra of mustard seeds after smoothing with SG (**a,b**), SNV and SG smoothing (**c,d**) and SG-D2 (**e,f**) to Vis–NIR and SWIR spectral data, respectively.

The average reflectance spectra (Fig. 1a,b) obtained for mustard seeds using Vis–NIR–SWIR HSI showed different shapes and reflectance intensity in line with the experimental spectral range. The effect of microwave treatment probably affected the seed reflectance intensity due to reduced enzyme activity and denaturation of some proteins in the mustard seeds^37^. It was observed that the variations in reflectance values among treatments were greater in the Vis–NIR spectral region than in the SWIR region. In Vis–NIR spectra with SG smoothing (Fig. 1a) the curves depicted a steep drop from 400 to 441 nm, it was observed that the variations in reflectance values among treatments were greater in the Vis–NIR spectral region than in the SWIR region. In Vis–NIR spectra with SG smoothing (Fig. 1a) the curves depicted a steep drop from 400 to 441 nm, followed by a gradual lowering up to 557 nm, then a steady increase was observed up to 1000 nm, which was predominantly caused by the third overtone of C–H stretching^38^ and relatively flat profile was observed with SNV and SG smoothing (Fig. 1c). In the case of the SWIR hyperspectral region SG smoothing (Fig. 1b) and SNV with SG smoothing (Fig. 1d) revealed prominent reflectance bands at 1140, 1164, 1374 and 1597 nm. The spectral range of about 1164 and 1374 nm^39^ relates to the C–H stretching vibration elongation in the second overtone (–CH_2_), which can be ascribed to the oil content^40^. Also, the O–H stretching in the first overtone is typically associated with water and its peak is observed at 1597 nm, in this case, it is likely that the peak at 1597 nm is related to cellulosic components instead of water due to the low moisture level of the samples (about 7%). Therefore, even though the peak is typically associated with water, in this specific case, it is more likely connected to cellulosic components. The performance of classification of the prediction models was bolstered by the presence of these peaks in the SWIR region, due to this the performance of the Vis–NIR HSI spectrum was lower than that of the SWIR HSI spectrum.

After performing an SG-D2 spectral pre-processing (Fig. 1e,f), additional peaks were observed throughout the spectral data. This phenomenon can be attributed to the inherent inverse relation between the amplitude and width of the successive derivatives of a wave-form; in this case it was the second derivative^41^. In Vis–NIR spectra which primarily represented the colour differences of the samples, the peaks and valleys were observed at 411 and 429 nm (violet band), 484 nm (blue band), 613 nm (yellow band) and 951, 990 nm^42^. The changes in the peaks and valleys can be attributed to the MW treatment of the mustard seeds, which may have caused alterations in their colour. In SWIR spectra, additional peaks and valleys were observed at 1160 and 1208 nm, representing the second overtone C–H. The observation of additional peaks and valleys in the C–H overtone region in the SWIR spectra suggests that MW treatment may have caused alterations in the chemical structure of the mustard seeds. These changes could be attributed to the heating effects of MW treatment, which may have caused the breakdown or rearrangement of chemical bonds^43^.

### Principal component analysis (PCA)

**Figure 2:**
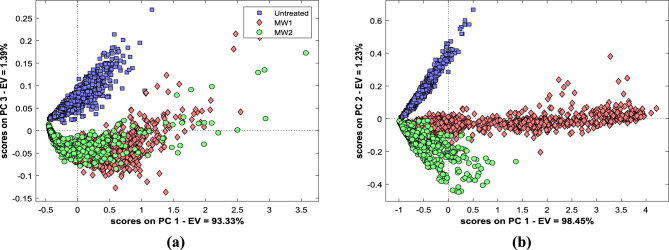
PCA for untreated and MW treated mustard seeds. PCA score plot of Vis–NIR (**a**) and SWIR (**b**) HSI pixel data, respectively.

The Vis–NIR–SWIR hyperspectral data was pre-processed and subjected to principal component analysis to depict variance across samples and to assess the influence of spectral pre-processing on the classification of MW-treated and untreated mustard seeds. The spectral data matrices for PCA contain 2700 reflectance data for each Vis–NIR and SWIR HSI samples. PCA model for the Vis–NIR–SWIR hyperspectral data is shown in Fig. 2.

In the score plot between PC1 and PC3 for Vis–NIR spectra (Fig. 2a), the difference in treated and untreated samples was clearly distinguishable, but it was difficult to segregate the samples amongst the treatments. The best possible separation of treated and untreated samples between negative and positive parts was observed in PC3. Untreated samples lie on the positive side and treated samples are mostly on the negative side of PC3. This type of cluster formation might be associated with variations in spectra due to the oil sample compositional discrepancies. This result shows that Vis–NIR HSI spectra can provide conclusive information to classify microwave-treated and untreated samples. In PCA loading plot of Vis–NIR, PC1 shows a dip at 417 nm; similarly, PC2 shows a peak at 417 nm and PC3 shows a peak at 441 nm, then loading curves in all PCs show no dips and peaks throughout the spectral region.

The plot score between PC1 and PC2 for SWIR spectra clearly demonstrates the difference between treated and untreated samples and also could differentiate amongst the treatments (Fig. 2b). The average distribution of untreated seeds was on the positive side, MW1 was distributed at zero scores, and MW2 was distributed on the negative side on PC2. It was observed that the treated MW1 seeds are on the positive side while treated MW2 and untreated seeds lie on the negative side of PC1. In PCA loading plots PC1 loadings showed fluctuation at 1082, 1379 and 1413 nm, PC2 loadings at 1082, 1364 and 1424 nm mainly related to carbohydrates and aliphatic chains of fats and protein. PC3 loadings show that 1135, 1374 and 1548 nm show variability in spectral regions, which is strongly influenced by aromatic compounds’ fatty acid chains^44^.

The loading plots PCA (see Supplementary Data, Fig. S1) indicate that the reflectance peaks observed in the spectra are similar to those in the plot and are primarily associated with fat components, water, cellulose molecules, or aromatic compounds.

**Figure 3:**
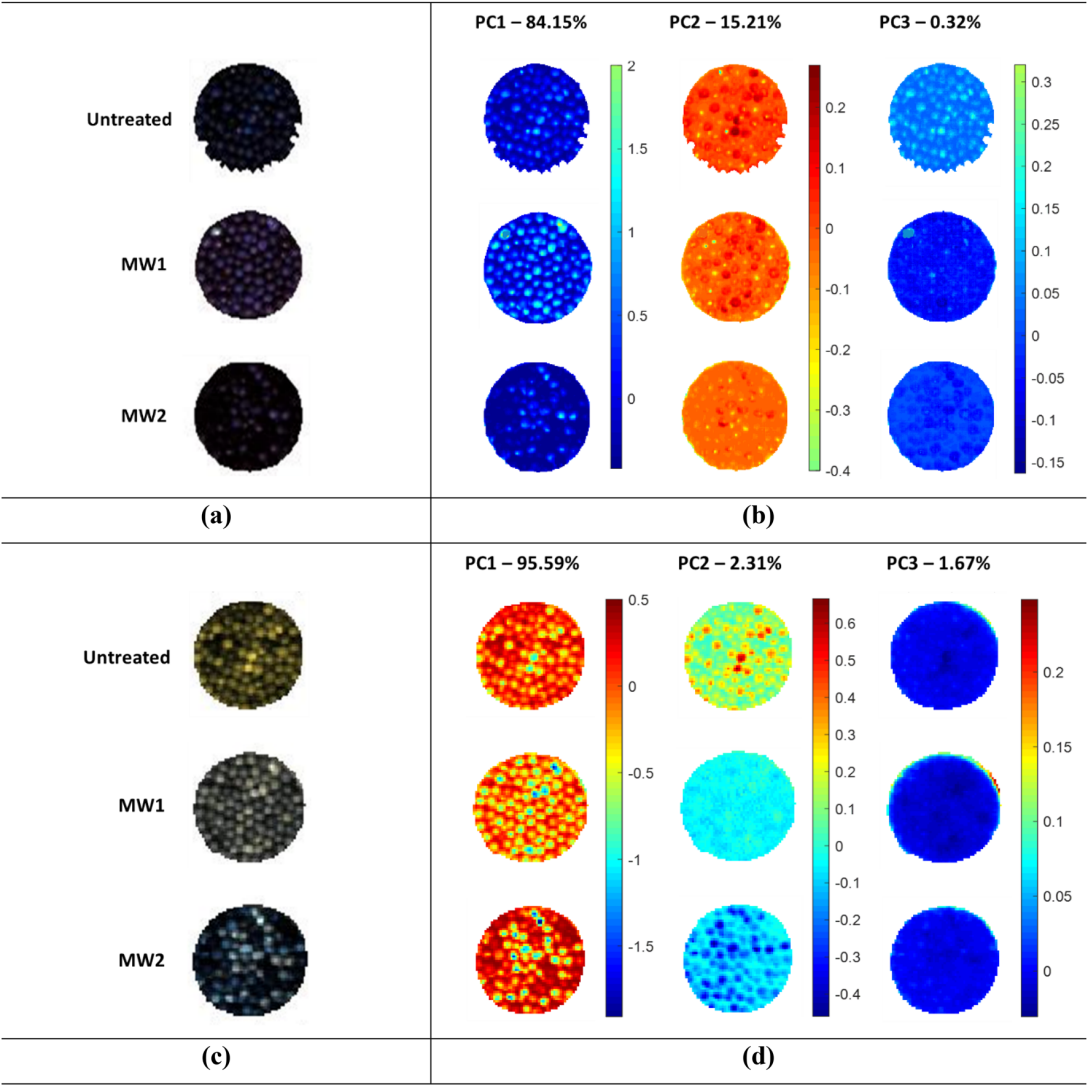
Hyperspectral images of Vis–NIR (**a**) and SWIR (**c**) HSI system. PC1 to PC3 score image obtained from Vis–NIR (**b**) and SWIR (**d**) HSI data.

The PCA score images obtained from hyperspectral images in the Vis–NIR–SWIR regions were analysed to identify differences between untreated and MW-treated samples based on hyperspectral images (Fig. 3a,c). The PCA score image of the Vis–NIR hyperspectral image (Fig. 3b) showed that PC1 exhibited a difference in contrast to the blue colour for the different samples, while PC2 and PC3 presented minimal changes in colour intensity. Conversely, for SWIR images (Fig. 3d) PC1 highlighted a considerable difference in contrast and intensity of red–orange–yellow pixels, while PC2 also displayed greater intensity and distribution of light blue–orange–blue pixels among the samples. Conversely, PC3 could not demonstrate any noticeable change. Observed differences in the PCA score images suggest that the SWIR region has a greater impact on spectral characteristics than the Vis–NIR region.

### Classification model development

PLS-DA models were developed for the classification of microwave treatment of mustard seeds using various spectral pre-processing methods with the best latent variables, and the performance was judged by indicators like sensitivity, specificity, precision, accuracy, and non-assigned data (Table 2).

**Table 2:** PLS-DA classification model performance for microwave pretreated mustard using SNV + SG smoothened Vis–NIR HSI spectral data and SNV + SGD2 smoothened SWIR HSI spectral data, with 10 and 3 latent variables, respectively.

	Calibration			Cross validation			Testing		
	Untreated	MW1	MW2	Untreated	MW1	MW2	Untreated	MW1	MW2
Vis–NIR									
Sensitivity	0.991	0.971	0.934	0.991	0.971	0.934	0.992	0.976	0.973
Specificity	0.995	0.964	0.990	0.995	0.964	0.989	0.998	0.984	0.989
Precision	0.991	0.936	0.976	0.991	0.936	0.976	0.996	0.972	0.974
Accuracy	0.966			0.966			0.981		
Non-assigned	0.116			0.116			0.087		
SWIR									
Sensitivity	1.000	0.998	0.990	1.000	0.997	0.989	1.000	0.984	1.000
Specificity	1.000	0.995	0.999	1.000	0.995	0.998	1.000	1.000	0.992
Precision	1.000	0.990	0.997	1.000	0.990	0.997	1.000	1.000	0.981
Accuracy	0.996			0.995			0.994		
Non-assigned	0.011			0.012			0.006		

The models developed using SWIR HSI spectra exhibited improved classification abilities compared to those created from Vis–NIR HSI spectra. SWIR HSI spectra demonstrated the higher sensitivity, specificity, and precision values, lower error rates, and greater accuracy. The spectral pre-processed SWIR HSI data also increases the classification ability of the models. The model trained with spectral data pre-processing SNV with SG-D2 yielded the best classification score, with sensitivity, specificity and precision values of more than 0.989, 0.995 and 0.990, respectively, with accuracy greater than 0.995 and non-assigned rate less than 0.012. In the case of Vis–NIR HSI spectra, the best classification score obtained from SNV with SG smoothing spectral pre-processing shows the strong capability to classify the different MW pre-treatment from control samples with a greater score of sensitivity from 0.934 to 0.991, specificity from 0.964 to 0.990 and precision from 0.936 to 0.991 with accuracy greater than 0.996 and non-assigned rate less than 0.116.

The high-performance level in the classifications of treated MW1 and MW2 seed samples in both Vis–NIR and SWIR HSI was due to small variations in time of exposure and bed thickness. The findings reported here in relation to classification performance of PLS-DA models using the Vis–NIR–SWIR HSI spectra are in line with that of wheat kernels^38^, muskmelon seeds^45^ and corn seeds^37^ as the subjects.

### Prediction model development

The PLS regression (PLS-R) models were developed to estimate the amount of oil and fatty acids in mustard seeds using a range of spectral pre-processing techniques^39^. Individual models were developed for Vis–NIR–SWIR, and the obtained RMSEC and RMSECV values, along with the number of latent variables used, were found to be satisfactory, indicating that the models were statistically adequate can be applied to independent test set (Table 3).

**Table 3:** Prediction of oil content and fatty acid contents in mustard seeds from PLSR model using Vis–NIR and SWIR HSI spectra.

	Spectral pre-processing	LV	Calibration		Cross validation		Testing			
			${R}_{C}^{2}$	RMSEC	${R}_{CV}^{2}$	RMSECV	${R}_{P}^{2}$	RMSEP	Bias (%)	Slope
Vis–NIR										
Oil content	SG smoothing + SG-D1	7	0.833	1.284	0.830	1.296	0.822	1.332	− 0.531	0.823
Fatty acids										
C18:1	SG-D1	6	0.839	0.185	0.836	0.187	0.842	0.184	0.086	0.831
C18:2	SNV	14	0.900	0.146	0.896	0.149	0.894	0.151	0.108	0.872
C20:1	SG smoothing	12	0.858	0.058	0.854	0.059	0.829	0.064	0.082	0.849
C18:3	SG smoothing	12	0.862	0.286	0.857	0.291	0.840	0.308	0.047	0.868
C22:1	SG smoothing	15	0.807	0.568	0.798	0.581	0.781	0.603	0.419	0.802
Saturated	SNV	12	0.878	0.135	0.873	0.138	0.871	0.140	0.075	0.857
Unsaturated	SNV	12	0.878	0.135	0.873	0.138	0.871	0.140	− 0.075	0.857
MUFA	SNV	18	0.713	0.449	0.698	0.460	0.723	0.433	0.775	0.655
PUFA	Raw	6	0.754	0.401	0.742	0.411	0.691	0.446	0.701	0.715
SWIR										
Oil content	SNV	10	0.995	0.223	0.994	0.231	0.993	0.267	0.090	0.972
Fatty acids										
C18:1	SNV + SG smoothing	11	0.994	0.041	0.992	0.042	0.994	0.041	0.029	0.965
C18:2	SNV	12	0.955	0.093	0.950	0.097	0.955	0.093	0.071	0.947
C20:1	SNV + SG-D2	8	0.964	0.030	0.962	0.031	0.946	0.036	− 0.052	0.944
C18:3	SNV + SG smoothing	12	0.972	0.132	0.971	0.134	0.957	0.160	0.003	0.931
C22:1	SNV + SG-D2	9	0.939	0.331	0.935	0.342	0.920	0.365	0.123	0.914
Saturated	SNV	12	0.994	0.028	0.994	0.030	0.949	0.087	− 0.122	0.973
Unsaturated	SNV	12	0.994	0.028	0.994	0.030	0.949	0.087	0.122	0.972
MUFA	SNV + SG-D2	9	0.903	0.262	0.895	0.272	0.899	0.264	0.046	0.887
PUFA	SNV + SG smoothing	13	0.915	0.243	0.913	0.247	0.857	0.304	− 0.107	0.895

*LV* latent variables, *R*^*2*^ coefficient of determination, *RMSEC* root mean square error for calibration, *RMSECV* root mean square error for cross validation, *RMSEP* root mean square error for testing.

In general, PLS-R models developed using SWIR spectra demonstrated better calibration and cross-validation capabilities as compared to Vis–NIR spectra. Furthermore, these SWIR-based models exhibit enhanced prediction abilities when applied to the external validation set. The study found that the best model for predicting oil content in mustard seeds was developed using SWIR data with SNV and SG smoothing spectral pre-processing. This model had high accuracy, with R^2^_C_, R^2^_CV_ and R^2^_P_ values of 0.995, 0.994 and 0.993, respectively; and a low error, with RMSEC, RMSECV and RMSEP values of 0.223, 0.231 and 0.267, respectively. The model also displayed relatively low bias (0.090%) and a high slope (0.972) in the testing set. In comparison, the Vis–NIR model developed with SG smoothing and SG-D1 spectral pre-processing had lower accuracy, with R^2^_C_, R^2^_CV_ and R^2^_P_ values of 0.833, 0.830 and 0.822, respectively; and higher error, with RMSEC, RMSECV and RMSEP values of 1.284, 1.296 and 1.332, respectively with the model had a bias of − 0.531% and a slope of 0.823 in the testing set. The PLS-R model exhibited a similar trend while predicting fatty acid components as well, the SWIR spectra of mustard seeds outperformed the Vis–NIR spectra. Deamination of fatty acid components in Vis–NIR data SNV with SG smoothing gave better accuracies with R^2^_C_ ranging from 0.713 to 0.900, R^2^_CV_ values ranging from 0.698 to 0.896. Furthermore, the R^2^_P_ values ranged from 0.691 to 0.894 and the bias remained below 0.775% with slope values greater than 0.715. These outcomes were achieved using 6 to 18 latent variables (LVs). Moreover, for SWIR data with SNV alone and SNV with SG-D2 spectral pre-processing gave higher accuracies ranging R^2^_C_ values from 0.903 to 0.994, R^2^_CV_ values from 0.895 to 0.994 and R^2^_P_ values ranging from 0.857 to 0.994 with LVs ranging from 8 to 12. Additionally, the bias remained consistently below 0.123%, indicating a negligible systematic deviation from the true values. The slope values, exceeding 0.887, indicated a strong linear relationship between the predicted and actual values. The saturated fatty acid was predicted with the highest accuracy by SWIR HSI system with SNV spectral pre-processing data with R^2^_C_, R^2^_CV_ and R^2^_P_ values of 0.994, 0.994 and 0.949, respectively; and RMSEC, RMSECV and RMSEP values were 0.028, 0.030 and 0.087, respectively. Similar findings have been reported by other researchers using Vis–NIR–SWIR HSI systems to estimate the oil content of peanuts^16^, brassica seeds^35^ and maize kernel^46^ and using NIR spectroscopy for coriander oil^47^.

In the study, the iPLS model was employed to identify the most useful wavelengths for predicting oil content in mustard seeds using Vis–NIR–SWIR spectra, with different spectral pre-processing procedures (Table 4). The results demonstrated that the iPLS model outperformed relevant, comprehensive models for oil content prediction, where superior performance was observed for Vis–NIR spectra and comparable performance for SWIR spectra. The performance for fatty acids prediction was also good except for C18:2 and MUFA (Vis–NIR) and C18:1 (oleic acid), C18:2 and PUFA (SWIR). It leads one to conclude that this model was able to predict the parameters without substantial performance loss.

**Table 4:** Prediction of oil content and fatty acid contents in mustard seeds from iPLS model using Vis–NIR and SWIR HSI spectra.

	Spectral pre-processing	NW	LV	Calibration		Cross validation		Testing	
				${R}_{C}^{2}$	RMSEC	${R}_{CV}^{2}$	RMSECV	${R}_{P}^{2}$	RMSEP
Vis–NIR									
Oil	SNV	14	3	0.877	1.139	0.877	1.140	0.748	1.585
Fatty acids									
C18:1	SNV	12	5	0.875	0.168	0.874	0.168	0.763	0.225
C18:2	SNV	20	9	0.659	0.281	0.653	0.283	0.645	0.275
C20:1	Raw	50	8	0.850	0.060	0.847	0.061	0.804	0.069
C18:3	SG smoothing	74	9	0.850	0.298	0.848	0.301	0.816	0.330
C22:1	Raw	48	8	0.771	0.619	0.767	0.625	0.784	0.598
Saturated	SNV	12	3	0.834	0.172	0.834	0.172	0.777	0.184
Unsaturated	SNV	12	4	0.836	0.171	0.835	0.172	0.777	0.184
MUFA	SNV	32	9	0.613	0.528	0.606	0.533	0.595	0.536
PUFA	Raw	64	12	0.725	0.424	0.716	0.431	0.691	0.446
SWIR									
Oil	SNV + SG-D2	72	6	0.884	1.060	0.880	1.076	0.885	1.058
Fatty acids									
C18:1	SNV + SG-D2	38	12	0.882	0.158	0.874	0.163	0.856	0.176
C18:2	SNV	12	4	0.780	0.208	0.777	0.209	0.715	0.247
C20:1	SNV	82	10	0.874	0.057	0.864	0.059	0.836	0.063
C18:3	SNV	84	9	0.871	0.283	0.863	0.291	0.866	0.288
C22:1	SNV + SG smoothing	74	12	0.861	0.501	0.856	0.510	0.861	0.501
Saturated	SNV	12	4	0.925	0.102	0.925	0.103	0.828	0.162
Unsaturated	Raw	26	7	0.821	0.157	0.820	0.158	0.802	0.174
MUFA	SNV	56	10	0.848	0.337	0.840	0.345	0.812	0.354
PUFA	Raw	32	8	0.793	0.381	0.791	0.383	0.753	0.399

*LV* latent variables, *NW* number of wavelengths, *R*^*2*^ coefficient of determination, *RMSEC* root mean square error for calibration, *RMSECV* root mean square error for cross validation, *RMSEP* root mean square error for testing.

The wavelengths identified using the iPLS model using an interval size of 2 for oil content prediction were 399–417, 770–815 and 860–866 nm with 3 LVs for Vis–NIR spectra, and 895–948, 962–976, 967–1020, 1034–1048, 1063–1068, 1082–1087, 1218–1223, 1237–1350, 1365–1370, 1384–1419, 1434–1498 and 1622–1627 nm with 6 LVs for SWIR spectra. For fatty acid components, the best iPLS model in case of Vis–NIR was predicted for C18:1 with 5 LVs wavelengths identified as 399–405, 770–789, 808–815 and 860–866 nm and saturated fatty acids in SWIR with 4 LVs wavelengths identified were 967–972, 986, 1306, 1320, 1419, 1498–1508, 1523–1528 and 1632 nm. It should be noted that the developed PLSR models may not be accurate when applied to external samples sourced from different varieties, harvest seasons, or were collected or stored in diverse environmental conditions^48–50^. To ensure practical usability, it is crucial to recalibrate the PLSR models presented in this study regularly. This can be achieved by using newly harvested and prepared samples that have been subjected to various pre- and post-harvest conditions. Most of the wavelengths that played a significant role in predicting the mustard oil and fatty acid content in both PLS and iPLS models were found to be similar to those observed in the HSI spectra and identified in the PCA loadings. Similar findings have been reported to predict the oil content in maize kernel^46^ and in various peanut varieties^51^.

**Figure 4:**
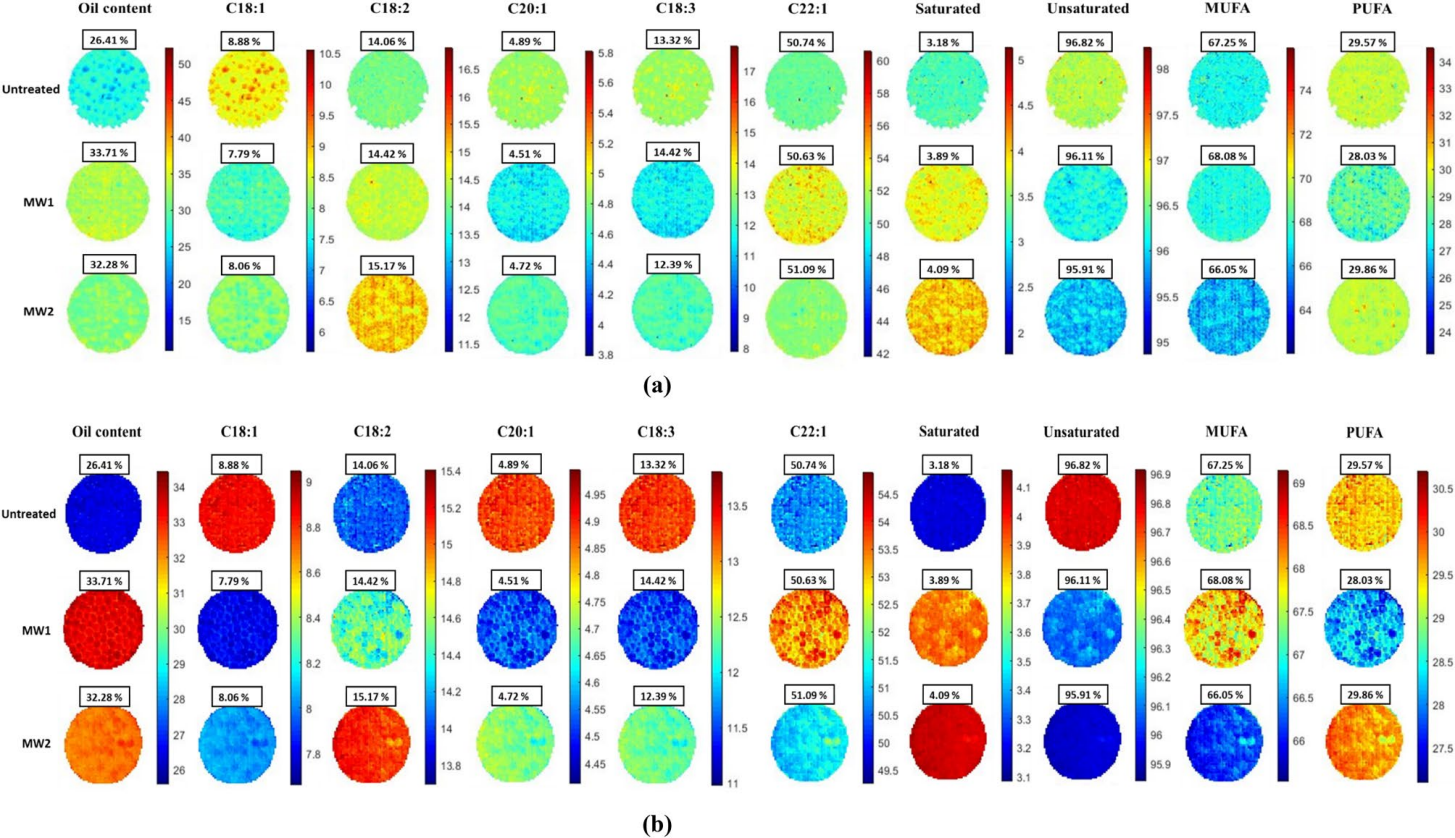
Prediction map of Vis–NIR (**a**) and SWIR (**b**) HSI imaging based on PLS-R model.

The PLS model was used to determine the spread of oil and fatty acid content across the seeds in a hyperspectral image. This model was applied across each pixel of a hyperspectral image to generate prediction maps illustrating the distribution of oil-related compositional components. To assess the effect of different MW pre-treatments on mustard seeds, prediction maps relating to the oil and fatty acid content were generated for each image (Fig. 4a,b).

## Conclusion

In this study, Vis–NIR–SWIR HSI systems together with associated chemometrics were used to quantify the oil content of microwave-treated mustard seeds and identify the fatty acid composition of mustard oil extracted from the seeds thereof. In comparison to Vis–NIR, the classification models created using SWIR HSI spectra, demonstrated better capabilities, with correct PLS-DA classification accuracy of 96.6 and 99.6%, respectively. The analytical results obtained by reference method used for these samples supported the predictions made for the oil and fatty acid content of mustard seeds.

In the prediction model, PLS-R models developed using SWIR spectra demonstrated better calibration, cross-validation and testing (R^2^_C_ = 0.995, R^2^_CV_ = 0.994 and R^2^_P_ = 0.994) capabilities and the iPLS model employed for prediction was also adequate. The accuracy of models to predict the oil and fatty acid content in microwave-treated mustard seeds was better for SWIR-HSI, particularly for saturated fatty acids and oleic acid. Spatial features studied using prediction maps revealed a significant benefit of using SWIR over Vis–NIR HSI. However, results obtained by Vis–NIR are quite acceptable and can be used for practical applications. Furthermore, the variable selection method allows the selection of important wavelengths to predict the parameters without a substantial performance loss; this leads us to conclude that Vis–NIR–SWIR HSI is an able tool to assess the quality and quantity of oil in the mustard seeds.

### Supplementary Information

Supplementary Figure S1.

## Data Availability

The datasets generated during and/or analysed during the current study are available from the corresponding author on reasonable request.
