# Supplementary material for: A chemometric approach to assess the oil composition and content of microwave-treated mustard (Brassica juncea) seeds using Vis–NIR–SWIR hyperspectral imaging
Source: Sci Rep. 2024 Jul 8;14:15643. doi: 10.1038/s41598-024-63073-0 (PMC11231289; doi:10.1038/s41598-024-63073-0)
Supplement: Supplementary file 1 — Supplementary Figure S1. [file 41598_2024_63073_MOESM1_ESM.docx]

**Supplementary Data:**

|  |  |
| --- | --- |
| **(a)** | **(b)** |

**Figure S1.** PCA loadings of Vis-NIR (**a**) and SWIR HSI (**b**) data, respectively.
